# Evaluation of the Diagnostic Performance of ChatGPT in Radiographic Staging of Sacroiliitis According to the Modified New York Criteria

**DOI:** 10.5152/ArchRheumatol.2026.25254

**Published:** 2026-01-16

**Authors:** Uğur Güngör Demir, Ali Nail Demir, Alper Uysal

**Affiliations:** 1 Department of Physical Medicine and Rehabilitation, Mersin City Training and Research Hospital, Mersin, Türkiye; 2 Department of Rheumatology, Mersin City Training and Research Hospital, Mersin, Türkiye

**Keywords:** Ankylosing spondylitis, artificial intelligence, ChatGPT, diagnostic accuracy, modified New York criteria, sacroiliitis

## Abstract

**Background/Aims:**

**:** This study aimed to evaluate the diagnostic performance of ChatGPT in grading sacroiliitis on pelvic radiographs according to the modified New York criteria.

**Materials and Methods:**

This retrospective study included 266 individuals with or without radiographic sacroiliac joint involvement according to the modified New York criteria (231 with ankylosing spondylitis and 35 without radiographic evidence of sacroiliitis). Two experts independently graded all radiographs based on the modified New York criteria, with disagreements resolved by a third reviewer. ChatGPT-5o (OpenAI, 2025) was prompted to classify each radiograph using a standardized English-language instruction. ChatGPT’s grading outputs were compared with expert consensus.

**Results:**

A statistically significant association was found between ChatGPT and expert gradings, but agreement remained slight (κ = 0.136). Multi-class performance was limited (overall accuracy = 30%), while binary analysis showed higher apparent accuracy (78%) due to a strong positive bias. Sensitivity was 0.796, specificity was 0.696, positive predictive value was 0.946, and negative predictive value was 0.338. Per-grade area under curve values ranged from 0.52 to 0.75, with the highest for Grade 0.

**Conclusion:**

ChatGPT demonstrated only limited agreement with expert assessments and showed poor ability to distinguish between sacroiliitis stages, performing adequately only for normal joints. These findings suggest that large language models like ChatGPT are unsuitable for direct radiographic interpretation without integration into specialized, vision-based diagnostic frameworks.

**Main Points**

- ChatGPT had only slight agreement with expert grades (κ = 0.136), and its classification of sacroiliitis stages was not reliable.
- One-vs-all and receiver operating characteristic analyses showed near-chance discrimination for Grades 1-4 and clear separation appeared only for Grade 0.
- In the binary evaluation (sacroiliitis present vs. absent), the model produced a high number of positive predictions, which led to 78% accuracy and a very high positive predictive value but a low negative predictive value.
- The overall findings show that a general-purpose language model cannot replace dedicated image-based artificial intelligence systems for radiographic assessment of the sacroiliac joints.

## Introduction

Sacroiliitis is a hallmark radiographic finding for the diagnosis of axSpA and ankylosing spondylitis (AS),^1^ and the assessment of sacroiliac joints by conventional pelvic radiographs remains a cornerstone in clinical investigation and disease classification.^2^ Although the modified New York criteria remain the most widely accepted system for grading sacroiliitis, it is limited by considerable interobserver variability, especially in early or borderline cases.^3^^-^^5^

Recent advances in artificial intelligence (AI), especially in deep learning, have significantly enhanced the automated detection and grading of sacroiliitis on pelvic radiographs.^6^^,^^7^ In addition, other AI-based systems improved the diagnostic and grading performance of junior radiologists by increasing consistency and accuracy during the assessment of sacroiliac joint changes.^7^

It is also important to note that most traditional medical information systems are targeted mainly toward medical professionals, whereas applications based on AI, like ChatGPT, directly provide patients with health-related information and personalized support.^8^^,^^9^

Till now, ChatGPT has been increasingly applied across a wide array of fields, including education, healthcare, engineering, and even the analysis of medical images. However, outputs from ChatGPT still have significant limitations regarding reliability, generalizability, and accuracy in medical imaging.^10^ Besides, systematic evaluations of its performance in this domain remain very rare. In this paper, an evaluation of ChatGPT’s performance regarding the grading of sacroiliitis on pelvic radiographs according to the modified New York criteria is presented. The current research does not aim to advocate for the use of ChatGPT as a clinical tool but rather assesses the current limitations of publicly available large language models when applied to radiographic reasoning tasks.

## Materials and Methods

### Study Design and Setting

This retrospective study was conducted at Mersin City Training and Research Hospital between October 22, 2025, and November 1, 2025. Ethical approval (Decision No.: 2025/07) was obtained from the Mersin City Training and Research Hospital Ethics Committee on October 20, 2025, before the initiation of data review. Data recorded in the hospital system between September 2023 and September 2025 were included in the study. Because this study was conducted retrospectively using anonymized radiographs, obtaining written informed consent from participants was not required.

### Study Population

The study included 231 patients diagnosed with AS according to the modified New York criteria and 35 healthy controls. A total of 266 anteroposterior pelvic radiographs were included after exclusions. Two radiographs contained a unilateral prosthesis; therefore, only the contralateral native joint was evaluated. Thus, a total of 530 sacroiliac joints were evaluated in the study. For each patient, only a single anteroposterior pelvic radiograph, which was the image with the highest diagnostic quality, was included in the analysis.

Inclusion criteria for the study were patients aged 18 years and older who had been diagnosed with AS according to the modified New York criteria and had anteroposterior (AP) pelvic radiographs, and healthy control groups with normal sacroiliac joints (Grade 0). For AS patients classified as Grade 1 on radiography, only those with additional magnetic resonance imaging (MRI) confirmation of sacroiliitis were included in the study, and all individuals in the control group (Grade 0) also underwent MRI to verify the absence of subclinical sacroiliitis.

Exclusion criteria comprised radiographs containing prostheses or foreign bodies, images with technical artifacts such as bowel gas overlap, pelvic rotation, or soft-tissue shadowing that rendered both sacroiliac joints uninterpretable, and cases with concomitant mechanical, infectious, malignant, or other rheumatologic diseases affecting the sacroiliac joints. Previous pelvic fractures, severe trauma, or degenerative changes that could alter the morphology of the sacroiliac joints, pregnancy, poor-quality or incomplete images, and patients younger than 18 years of age were other exclusion criteria. However, if at least 1 sacroiliac joint was clearly assessable, the radiograph was retained, and equivocal or Grade 1 cases were verified with sacroiliac joint MRI to minimize misclassification.

### Image Acquisition and Preparation

The images used in this study were standard AP pelvic radiographs, which represent the primary radiographic technique recommended by the modified New York criteria for assessing sacroiliitis.^11^ All AP pelvic radiographs were retrieved retrospectively from the hospital’s Picture Archiving and Communication System (Simplex PACS, Türkiye). Images were exported in JPEG format with a standardized resolution of 512 × 512 pixels. All images were fully anonymized and stripped of metadata prior to upload to ensure patient data protection.^12^

### Manual Reference Grading

Two experienced clinicians, 1 physical medicine and rehabilitation specialist (U.G.D.) and 1 rheumatologist (A.N.D.), independently graded all radiographs using the modified New York criteria. In cases of disagreement, a third physical medicine and rehabilitation specialist (A.U.) served as the adjudicator. The radiographic grading of sacroiliitis according to the modified New York criteria includes 5 stages, ranging from Grade 0 to Grade 4. Grade 0 represents a normal sacroiliac joint with no pathological findings. Grade 1 indicates suspicious changes such as minimal sclerosis that may suggest early involvement. Grade 2 corresponds to definite but mild abnormalities characterized by evident sclerosis, minimal erosions, and slight joint-space narrowing. Grade 3 reflects a moderate degree of abnormality that shows marked sclerosis, clear erosions, and partial ankylosis. Finally, Grade 4 represents complete ankylosis with total obliteration of the sacroiliac joint space.^13^ Patients with AS who were classified as stage 1 based on radiographic findings were additionally confirmed by MRI before inclusion in the AI analysis. Interobserver reliability between A.N.D. and U.G.D. was assessed using Cohen’s kappa, with a value of 0.68 interpreted as substantial consistency.

### Artificial Intelligence–Based Evaluation

Radiographic grading by AI was performed using ChatGPT-5o (OpenAI, 2025; https://chatgpt.com/). A dedicated, newly created account was used to ensure independence from prior interactions.^9^ Each radiograph was uploaded separately and analyzed using the standardized English-language prompt: “Please evaluate this anteroposterior pelvic radiograph according to the modified New York grading system.” ChatGPT-5o was used with a multimodal configuration that allows for visual input interpretation. The model operates through linguistic and contextual reasoning rather than true pixel-level image analysis; therefore, its visual assessments represent descriptive rather than algorithmic processing. Default system parameters were used without any fine-tuning or temperature modification to maintain methodological consistency and reproducibility. The AI model’s classification outputs were then compared with the consensus expert grades.

### Statistical Analysis

All statistical analyses were performed using IBM SPSS Statistics, version 24.0 (IBM SPSS Corp.; Armonk, NY, USA). The diagnostic performance of ChatGPT in grading sacroiliitis on pelvic radiographs was evaluated through a series of categorical agreement and classification analyses. Cross-tabulation analysis was applied to compare ChatGPT’s 0-4 grading outputs with expert reference grades. Pearson’s chi-square test was used to determine the association between the 2 rating systems. Cohen’s kappa coefficient quantified the inter-rater agreement beyond chance. Per-grade diagnostic performance was calculated using a 1-vs-all approach, where each grade (0-4) was treated as a separate binary classification (target grade vs. all others). For each grade, positive predictive value (PPV), sensitivity, specificity, and accuracy were computed using the standard formulas. Diagnostic performance metrics were calculated to assess the agreement between ChatGPT and expert evaluations. The following indicators were computed for each sacroiliitis grade: PPV, sensitivity, specificity, accuracy, and negative predictive value (NPV). Positive predictive value was defined as the ratio of true positives (TPs) to the total number of positive predictions, calculated as TP/(TP+FP). Sensitivity represented the proportion of correctly identified positive cases, given by TP/(TP+FN). Specificity measured the proportion of correctly identified negative cases, expressed as TN/(TN+FP). Accuracy indicated the overall proportion of correct classifications, computed as (TP+TN)/N. Negative predictive value was defined as TN/(TN+FN). Here, TP refers to correctly identified positive cases, false positive (FP) refers to cases incorrectly labeled as positive, false negative (FN) refers to cases incorrectly labeled as negative, and true negative (TN) refers to correctly identified negative cases. N refers to the total number of evaluated radiographs.^9^^,^^14^^,^^15^

Aggregated performance metrics (micro-, macro-, and weighted-average precision/sensitivity/specificity/accuracy) were derived to assess overall classification quality and to account for class imbalance. For the binary analysis (presence vs. absence of sacroiliitis), a 2 × 2 Crosstabs comparison was performed between the expert diagnosis and ChatGPT’s binary prediction. From this table, sensitivity, specificity, PPV, NPV, accuracy, and Cohen’s kappa were computed. Receiver operating characteristic (ROC) curve analysis was planned for both the multi-class (1-vs-all) and binary evaluations. The area under the curve (AUC) and 95% CIs were calculated for each grade and for the aggregated metrics (micro, macro, weighted averages). A two-tailed *P*-value of <.05 was considered statistically significant for all analyses.

## Results

A total of 266 anteroposterior pelvic radiographs were included after exclusions. Expert and ChatGPT gradings of sacroiliitis (modified New York 0-4) were compared. As shown in Table 1, although a statistically significant association was observed between ChatGPT and expert gradings of sacroiliitis (*Χ*^2^ = 101.07, *P* < .001), the inter-rater agreement remained slight (Cohen’s k = 0.136). As illustrated in Tables 2 and 3, the subsequent per-grade and aggregated performance analyses provided detailed precision, sensitivity, specificity, and accuracy metrics across all grading levels.

As presented in Table 4, the binary classification analysis yielded an accuracy of 78.3%, with a sensitivity of 0.796, specificity of 0.696, a PPV of 0.946, and a NPV of 0.338. Cross-tabulation of sacroiliitis presence showed a significant association (*Χ*^2^ = 73.993, *P* < .001), with a Cohen’s κ value of 0.339 (*P* < .001).

**Figure 1.**
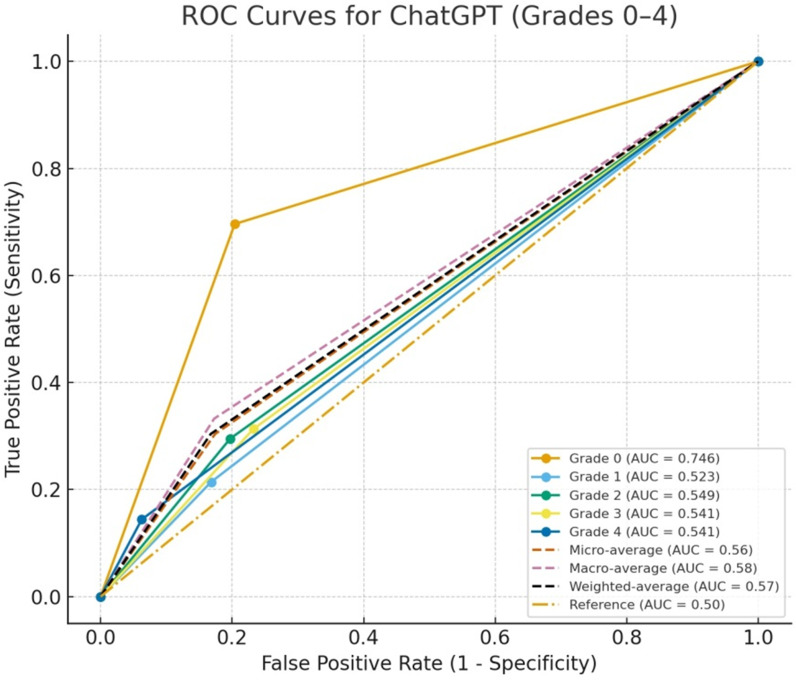
Receiver operating characteristic curves showing ChatGPT’s performance in grading sacroiliitis based on the modified New York criteria.

Receiver operating characteristic curve analysis revealed that ChatGPT had limited class-based discrimination. Grade 0 provided the highest discrimination with an AUC of 0.746, while Grades 1-4 had AUC values ranging from 0.523 to 0.549. The AUC values of Grades 1-4 demonstrated near-chance performance. Furthermore, the macro-average AUC was 0.58, the support-weighted average AUC was 0.57, and the micro-average AUC was 0.56; all values were close to the baseline (AUC = 0.50) (Figure 1). Meaningful separation appeared only for Grade 0, and Grades 1-4 remained close to the reference line in the ROC analysis.

## Discussion

In this study, the diagnostic performance of ChatGPT in grading sacroiliitis on pelvic radiographs was evaluated. Although ChatGPT’s predictions showed a statistically significant association with expert gradings, the low inter-rater agreement demonstrates that this relationship does not translate into clinically meaningful consistency. The findings demonstrated that the model’s discrimination capacity remains limited across most sacroiliitis grades. Consistent with the ROC analysis, ChatGPT showed relatively better ability to distinguish normal sacroiliac joints, but its performance on Grades 1-4 remained close to the random classification level. The macro-average, micro-average, and weighted-average AUC values were likewise close to the reference line, showing the model’s overall limited predictive accuracy. These results demonstrate the inadequacies of applying general-purpose large language models to detailed radiographic interpretation tasks. The binary accuracy of 78% was related to the high number of positive predictions, which produced a very high PPV but a low NPV and did not reflect balanced diagnostic performance.

The confusion matrix also shows that ChatGPT assigned higher grades more frequently, which explains the shift toward positive results in the binary analysis. AI represents a transformative advancement poised to reshape the field of radiology. By enabling novel analytical capabilities, AI holds great promise for enhancing both the efficiency and accuracy of medical image interpretation and leads to more effective clinical decision-making.^16^ Beyond simple automation, AI systems can assist radiologists in detecting subtle imaging patterns, reducing diagnostic errors, and optimizing workflow efficiency.^17^ It facilitates the automated assessment of disease progression, supports preoperative planning, and provides real-time assistance during minimally invasive surgical procedures.^18^ However, despite these advances, concerns remain regarding data quality, model transparency, and generalizability across diverse patient populations.^17^^,^^19^

Artificial intelligence applications have shown promise across various musculoskeletal imaging domains, including fracture detection, bone age estimation, osteoarthritis grading, tumor characterization, and implant assessment, and have helped radiologists improve diagnostic efficiency and accuracy.^20^

Building upon these advancements, recent research has explored the potential of AI in evaluating the sacroiliac joints, particularly for the automated detection and grading of sacroiliitis based on the modified New York criteria. Meng et al^7^ developed a ConvNeXt-T–based deep learning model that automatically graded radiographic sacroiliitis on pelvic X-rays. In the external test set, the model achieved a multi-class grading accuracy of 63.9% across all 5 stages and a diagnostic accuracy of 90.1% for detecting definite radiographic sacroiliitis. The AI system also enhanced the grading performance of junior radiologists. The data of their study indicate that AI assistance can improve both efficiency and consistency in sacroiliitis evaluation.^7^

Similarly, Lee et al^6^ proposed a ResNet18-based deep learning approach using magnetic resonance (MR) images to detect bone marrow edema in patients with axial spondyloarthritis. Their model achieved 93.6% accuracy in identifying edema on individual MR slices and 96.1% accuracy in diagnosing active sacroiliitis at the subject level. Their work has shown that deep learning applied to MRI analysis can be a reliable aid for clinicians in early disease diagnosis.^6^

Complementing these MRI-based findings, Fu et al^21^ employed multiple convolutional neural network architectures, including ResNeXt-50 and Inception-v4, for classifying computed tomography (CT) images of sacroiliitis and achieved up to 99% diagnostic accuracy. Their study emphasized not only the superior performance of these models but also their interpretability through Grad-Class Activation Mapping (CAM) visualization, which highlighted anatomically relevant regions corresponding to the sacroiliac joints.^21^

Collectively, these studies highlight the growing application of AI across various imaging modalities, including radiographs, CT, and MRI, particularly in the diagnosis and grading of sacroiliitis. These advancements underscore AI’s potential to automate and enhance diagnostic processes. However, the majority of these systems rely heavily on structured image data, such as pixel-level analysis and feature extraction, to perform tasks like disease classification and grading. In contrast, large language model–based tools like ChatGPT utilize linguistic and contextual reasoning, processing textual descriptions and other unstructured data, rather than engaging with raw image data directly. This fundamental difference in approach raises critical questions about the diagnostic reliability of such systems when applied to radiographic interpretation.^22^ While ChatGPT’s capabilities in natural language understanding are impressive, further evaluation is required to assess its effectiveness and accuracy in medical imaging tasks that traditionally rely on detailed visual data analysis. Horiuchi et al^23^ reported that GPT-4-based ChatGPT, when using textual imaging descriptions, achieved diagnostic accuracy comparable to that of a radiology resident, whereas the image-based GPT-4V model performed markedly poorly. Similarly, Temel et al^9^ evaluated the performance of ChatGPT-4o on Kellgren–Lawrence grading of knee radiographs and found overall low diagnostic accuracy and nearly random AUC values. They found ChatGPT to have limited discriminatory capacity for radiographic interpretation tasks.^9^ In line with these observations, the present study also found that ChatGPT’s radiographic grading of sacroiliitis showed only slight agreement with expert evaluation. It has demonstrated that this shows consistency at almost a chance level and further supports the existing evidence that large language models are not reliable for direct image-based diagnosis assessment.

These findings not only illustrate the technical limitations of large language models in visual reasoning but also bring to mind broader challenges associated with their rapid integration into clinical and public domains. While many medical AI systems are restricted to clinical or institutional use, ChatGPT and similar models are publicly accessible and increasingly used in medical contexts. However, their open accessibility raises concerns about data privacy, ethical use, and self-diagnosis/diagnostic reliability.^9^^,^^24^ To ensure the safe and effective implementation of ChatGPT and similar AI tools, further refinement under expert supervision and validation with high-quality datasets is essential. Although not a clinical tool, the findings bring to mind the risks associated with inaccurate information when using unvalidated, publicly available large language models such as ChatGPT in medical image analysis and underscore the need for regulatory oversight.

This study has some limitations. Its retrospective, single-center design and small control group may limit generalizability. Image quality and acquisition parameters varied across radiographs, which might have affected grading accuracy. Moreover, ChatGPT is a general-purpose language model without direct image-analysis capability, so its diagnostic performance cannot be compared with dedicated medical AI systems.

In summary, ChatGPT showed only limited alignment with expert assessments and was unable to reliably discriminate between different stages of sacroiliitis. Although it performed somewhat better in identifying completely normal joints, its accuracy substantially declined across early and advanced disease categories, with frequent misclassifications in both per-grade and binary evaluations. These findings suggest that current large language models, including ChatGPT, lack true radiographic reasoning ability and should not be used for direct diagnostic interpretation of medical images. Future research should focus on integrating language models with domain-specific visual learning frameworks and validating such systems under expert supervision to ensure safe and clinically meaningful implementation in medical imaging.

## Figures and Tables

**Table 1.** Confusion Matrix for ChatGPT vs. Expert Sacroiliitis Grades

**Expert Grade**	**Prediction 0**	**Prediction 1**	**Prediction 2**	**Prediction 3**	**Prediction 4**	**Total**
0	48	10	5	4	2	69
1	10	9	5	16	2	42
2	35	24	41	28	11	139
3	30	25	28	42	9	134
4	19	23	39	44	21	146
Total	142	91	118	134	45	530

Pearson’s *χ*^2^ = 101.07, *df* = 16, *P* < .001.

Cohen’s κ = 0.136.

**Table 2.** Per-Grade Diagnostic Performance (One-vs-All Analysis)

**Grade**	**Precision**	**Sensitivity**	**Specificity**	**Accuracy**
0	0.338	0.696	0.796	0.783
1	0.099	0.214	0.832	0.783
2	0.347	0.295	0.803	0.670
3	0.313	0.313	0.768	0.653
4	0.467	0.144	0.938	0.719

**Table 3.** Aggregated Performance Metrics

**Metric**	**Micro-Avg**	**Macro-Avg**	**Weighted-Avg**
Precision	0.304	0.313	0.351
Sensitivity	0.304	0.332	0.304
Specificity	–	0.827	0.833
Accuracy	0.304	0.722	0.703

Avg, average.

**Table 4.** Binary Classification (Presence vs. Absence of Sacroiliitis)

**Metric**	**Value**
Sensitivity	0.796
Specificity	0.696
Positive predictive value	0.946
Negative predictive value	0.338
Accuracy	0.783

## Data Availability

The data that support the findings of this study are available on request from the corresponding author.
